# Understanding IV antimicrobial drug losses: the importance of flushing infusion administration sets

**DOI:** 10.1093/jacamr/dlaa061

**Published:** 2020-08-11

**Authors:** Balwinder Bolla, Yeshmita Buxani, Rebecca Wong, Leslie Jones, Michelle Dube

**Affiliations:** United Lincolnshire Hospitals NHS Trust, Lincoln County Hospital, Greetwell Road, Lincoln LN2 5QY, UK

## Abstract

**Background:**

IV drugs are commonly prescribed for inpatient treatment. Where administered as infusions, drug dose loss is incurred if the infusion line is not flushed. Underdosing of IV antimicrobials is of particular concern as reduced treatment efficacy increases the risk of patient deterioration (including sepsis) and development of antimicrobial resistance.

**Objectives:**

To quantify drug loss, raise awareness and provide recommendations to address this patient safety risk effectively.

**Methods:**

Percentage drug loss of 39 IV antimicrobials was calculated for a theoretical patient case scenario, using residual volumes for IV infusion lines utilized within this acute healthcare setting. An adult male patient (70 kg) with good renal function was assumed for drug dosing. Infusion volumes and doses are based on a widely used IV administration guide.

**Results:**

Data revealed the scope and extent of antimicrobial drug losses where infusion lines were not flushed as ranging from 2% to 33%. More than 10% of the drug would be lost for 26 of the 39 antimicrobials assessed, with five of these yielding over 20% loss.

**Conclusions:**

The authors suggest that unintentional antimicrobial underdosing is going unnoticed in clinical practice. Where IV infusion is necessary, flushing of the infusion line to ensure total dose administration is strongly recommended. Risks associated with flushing lines (fluid overloading, bolus dosing, etc.) can be managed with simple measures. The authors call for a national body-led approach to effectively influence healthcare organizations in review of IV administration protocols, ensuring patient safety and care in the NHS.

## Introduction

Critical medications are commonly administered IV in the acute hospital setting.^1^ It is the responsibility of healthcare professionals involved in drug administration to ensure that the correct therapeutic dose is administered.^1^^,^^2^ Underdosing of IV medications as a result of not flushing infusion lines is a potentially widespread issue that has only recently come to light and is now gaining national attention, with a need to expand the current state of knowledge and evidence base.^3^^,^^4^ The main concerns are around therapy failure and deterioration of the patient, with antimicrobial underdosing also potentially risking resistant organisms emerging.^2^ Whilst the risk of underdosing medications may be linked to the route of administration, the National Patient Safety Agency (NPSA) has specifically highlighted the increased risk linked to IV drug administration as a concern.^1^^,^^4^ The NPSA also reported that between January 2005 and June 2006, almost a quarter of all medication incidents related to injectable medicines.^1^

It is unclear why IV infusion lines are not commonly flushed in practice. Potential motivations include having not understood the risks to medicine optimization and safety, amidst a desire to save time and costs.^2^ There is also limited guidance in the UK surrounding the need to flush IV administration sets.^3^^,^^4^

The authors of this study set out to quantify the antimicrobial drug loss percentage in the absence of flushing the residual volume of the infusion set. Practical measurement on a clinical ward setting revealed the volume of drug discarded in the two different administration sets that are utilized in this acute healthcare setting.

The aim of this study was to assess the extent of IV antimicrobial drug loss in the IV infusion lines, and whether this would qualify a change in working practice policy to enforce flushing of IV infusion lines.

The objective of this study was to reveal, for a hypothetical patient case scenario, the percentage of antimicrobial drug loss in IV infusion lines which aren’t flushed, in order to understand the significance of this percentage loss in terms of risk to patients, and to consider potential solutions to the unintentional drug loss.

## Methods

A clinical pharmacist and a medical prescriber were assigned to undertake this study. An investigation into the most common IV infusion lines utilized on ward areas in our acute NHS trust was undertaken through the central procurement team. Name, manufacturer and length of infusion set line were derived from the product packaging. Residual volumes were obtained in-house from an average of three measurements in clinical practice.

A table was drawn up for all IV antimicrobials used by the trust and included dose assumptions that were drawn from the local antimicrobial guidelines,^5^ based on a theoretical patient case scenario: a 50-year-old male patient weighing 70 kg, with good renal function (CL_CR_ ≥ 90 mL/min) and no fluid restrictions.^6^

A trustwide reference source for the administration of IV antimicrobials was used to provide information on reconstitution, dilution and displacement values, to enable calculation of resulting infusion volumes and concentrations.^5^

Utilizing concentration values and the average residual volume of the infusion line, the drug loss was calculated in milligrams and then converted into percentage drug loss.

Table 1 shows the number of IV antimicrobials affected in respective percentage dose-loss ranges. The drug losses, as a percentage of the dose stated, for all IV antimicrobial drugs are listed in Table 2, which includes concentration of the infusion (in mg/mL) for reference.

**Table 1.** Number of antimicrobials in each percentage drug loss band, following IV infusion via the 180 and 235 cm infusion sets, respectively^7^^,^^8^

Percentage antimicrobial dose loss	Number of IV antimicrobials affected	
	180 cm infusion set	235 cm infusion set
0–5	11	4
6–10	14	9
11–15	9	15
16–20	4	6
21–25	1	1
>25	0	4

**Table 2.** Percentage of antimicrobial dose lost following an IV infusion, where the infusion line was not flushed, for the 180 and 235 cm infusion sets, respectively^7^^,^^8^

Antimicrobial	Dose assumed (mg)	Concentration of infusion (mg/mL)	Drug loss percentage	
			short line (180 cm)	long line (235 cm)
Aciclovir	700	2.52	4	6
Amikacin	500	4.90	11	16
Amoxicillin	1000	8.28	9	13
Anidulafungin	200	1.25	7	10
Aztreonam	2000	18.87	11	15
Benzylpenicillin	1200	9.93	9	13
Cefotaxime	2000	18.18	10	14
Ceftaroline	600	5.00	9	13
Ceftazidime	2000	33.33	19	26
Ceftobiprole	500	1.92	4	6
Ceftriaxone	2000	28.57	16	23
Cefuroxime	1500	12.92	10	14
Chloramphenicol	1700	14.53	10	14
Ciprofloxacin	400	2.00	6	8
Clarithromycin	500	1.92	4	6
Clindamycin	900	9.00	11	16
Co-amoxiclav	1200	10.00	9	13
Co-trimoxazole	1440	2.88	2	3
Daptomycin	420	7.19	19	27
Ertapenem	1000	16.67	19	26
Erythromycin	500	4.17	9	13
Flucloxacillin	2000	14.29	8	11
Fosfomycin	4000	40.00	11	16
Gentamicin	350	3.22	10	15
Imipenem/cilastatin	1000	4.16	5	7
Isavuconazole	200	0.78	4	6
Levofloxacin	500	5.00	11	16
Linezolid	600	2.00	4	5
Meropenem	2000	14.29	8	11
Metronidazole	500	5.00	11	16
Moxifloxacin	400	1.60	5	6
Piperacillin/tazobactam	4500	36.54	9	13
Rifampicin	600	1.18	2	3
Tedizolid	200	0.78	4	6
Teicoplanin	600	5.67	11	15
Temocillin	2000	41.67	24	33
Tigecycline	50	0.48	11	15
Tobramycin	70	0.69	11	16
Vancomycin	1500	4.55	3	5

### Ethics

This study used a theoretical patient case scenario, rather than real patient data. Therefore it does not classify as research under the UK Policy Framework for Health and Social Care and hence National Research Ethics Service review was not required.

## Results

The acute NHS trust was found to be utilizing two infusion-giving sets. The most commonly used set was the Intrafix Safeset. This is a gravity drip infusion set, manufactured by B. Braun Medical Ltd, which is 180 cm long. Average residual volume was measured to be 11.33 mL (range 10.82–12.11 mL).^7^ The other line was the Volumat VL ST 02, manufactured by Fresenius Kabi Ltd. This infusion set is 235 cm long and designed for use with rate-controlling pumps. Average residual volume was measured to be 15.83 mL (range 15.06–16.47 mL).^8^

The calculated drug losses based on the residual volumes determined above, in conjunction with the concentration of the infusion determined from the trustwide reference source,^5^ are presented in Tables 1 and 2.

There is positive correlation between antimicrobial concentration of the infusion prepared and the percentage drug loss.

## Discussion

This study does have several limitations due to being based on calculations for a hypothetical patient case scenario, rather than data from actual patient cases. This was an intentional feature to allow standardization and reporting of results, as the alternative of utilizing individual patient data would not have this benefit. One of the first limitations realized during measurement of the infusion lines was that the residual volume can be variable and is likely to be influenced by individual administration technique. Hence, the results are simply providing an approximation of drug loss. To be meaningful, a study comprising actual patients would require a large cohort to provide statistical significance and most likely require funding to provide resources for the labour-intensive data collection, as well as being conducted over a suitably long timescale to gather the number of cases required. This study does not have the ability to reflect variation in local or individual practice in regard to the method of administration, nor can it take account of individual technique in handling the infusion set, which would also be an important variable.

Furthermore, all weight-based calculations for this hypothetical case scenario assumed a body weight of 70 kg, with no renal impairment or fluid restriction.^6^ These assumptions result in further limitation in that it does not reflect drug dose adjustment, or infusion volume variation, at extremes of body weight, various degrees of renal impairment, or where fluid restrictions are in place.^2^^,^^9^ For the latter, the concentration of infusion may be much higher than that which is licensed as such patients often require the antimicrobial infusion volume (including flush) to be tailored into their daily fluid allowance.^5^ There is also limitation in dosing and drug loss assumed, as very severe and deep-seated infections would potentially warrant higher doses (and thereby higher concentration in the infusion bag) of some antimicrobials.^5^^,^^9^

A study conducted by Cooper *et al.*^3^ highlights the frequency at which intermittent IV infusions are underdosed due to not flushing the administration set. It puts forward that 5%–20% of IV drugs are left in the tubing of the IV giving sets after the administration of the IV drug, if the infusion line itself is not flushed, and underdosing of IV antimicrobials is a common feature.^3^

This study, specifically focusing on IV antimicrobials with dose assumptions made on a standardized body weight, renal function, age etc.,^6^ finds that in practice, percentage loss can be as much as 33% with the 235 cm line. It is likely that extremes of body weight would create further issues, with complications around volume of distribution and tissue penetration causing further concerns. Patients who are fluid restricted would also suffer greater drug losses and the repercussions for clinical deterioration are further complicated.^3^

There is certainly a loss of antimicrobial dose where infusion lines are not flushed.^3^ However, the extent of antimicrobial drug loss is far greater than was anticipated when this study was first constructed. Finding two infusion lines in mainstream use, the study confirms and elaborates on use of the longer 235 cm infusion set resulting in a greater drug loss in comparison with the shorter 180 cm infusion set (see Tables 1 and 2). Manufacturers of the giving sets do not highlight any difference in drug or diluent compatibility between the two products, so the choice is often based on the setting (i.e. ward versus operating theatres) and also on intended treatment. Longer infusion sets are used with infusion pumps, so the 180 cm infusion set appears to be the default option where IV infusion is needed.

The significant percentage of drug loss indicates the importance of reducing this impact.^1^ Consequences of unintentional dose reduction include risk of not attaining the minimum inhibitory concentration (MIC) required to treat the infection, longer time to achieve the required serum levels and deterioration in clinical condition, which may lead to sepsis,^1^ hence affecting morbidity and mortality, with little justification left for not administering the full dose prescribed.^1^^,^^2^ Ethical considerations need to be made for duty of candour, where a significant proportion of a prescribed antimicrobial dose is not administered.^2^ This could potentially be challenged in a court as a Medicines Act 1968 breach, if a patient were to experience a safety incident. With a prescription clearly stating a dose, any percentage drug loss should be questioned, especially where feasible solutions to prevent these losses (and the consequential issues associated with the antimicrobial drug loss) exist.^2^^,^^9^

The awareness of, and risks associated with, underdosing of IV medication has become a concern across various hospitals and is rapidly gaining national attention. The consequences of underdosing have not been fully researched in terms of efficacy and acute healthcare organization attempts to minimize the theoretical risk need to be collated nationally.

The risks of flushing lines may include fluid overload. However, this can be mitigated by limiting the volume of flush to a nominal amount that will cover the residual volume of the infusion line.^4^ Given that the residual volumes for both lines assessed were over 10 mL but under 20 mL, using a 20 mL flush would pose minimal risk of fluid overloading, especially for patients receiving multiple antimicrobial infusions per day. In effect, a 20 mL flush would simply replace the residual drug volume, which is not otherwise administered, with up to 9 mL surplus. Care must be taken in choosing the appropriate solution for flushing, with reference to the appropriate guidelines (i.e. Medusa).^5^ This recommendation is supported by The Marsden Manual of Clinical Nursing Procedures, where section 12.25 ‘Medication: intermittent infusion of IV drugs’ recommends the disconnection of the infusion set followed by flushing of the device with 0.9% sodium chloride or an alternative compatible solution.^10^

There is also risk of the residual infusion being given as a bolus when flushed through.^4^ This will be a concern with some antimicrobials and have implications for other critical medicines too.^9^ The current method of flushing infusion lines is to attach a small infusion bag of a suitable solution for flushing (i.e. sodium chloride 0.9%) to the original fluid bag, so it runs through at the same rate (going through the gravity chamber or infusion pump) as the main bulk of the dose did.^4^ Hence, this risk is mitigated with this method, but must be considered with any proposals for change in method of flushing infusion lines.^4^

It is important to highlight the ambiguity of the term ‘flush’, as recent attention on IV drug losses reveals that there are two distinct types of flush: the small-volume flush through a cannula and a larger-volume flush used for the length of tubing following an infusion. Establishing a nationally approved terminology for each type of flush, such as ‘a cannula flush’ and ‘an infusion-line flush’, to distinguish the difference, would improve awareness and enhance clarity rather than simply referring to a ‘flush’ alone.

The authors found that IV infusion lines in this acute NHS organization are routinely flushed on the oncology wards, paediatric wards and ICUs, whereas general surgical and medical wards do not routinely flush infusion lines. The study by Cooper *et al.*^3^ also found chemotherapy areas flush infusion sets, but other areas do not.^3^ With such practice already being implemented for vulnerable patient areas, such practice should be embedded as best practice for patient safety in all clinical settings.

In order to minimize drug dose losses, the authors make several recommendations, as follows. In the first instance, on a local level, there should be consideration of opting for IV bolus injection for antimicrobials, where appropriate, unless there is strong clinical reason not to.^5^^,^^9^ Where infusion is necessary, staff administering the medication should be encouraged to utilize the short line, unless the infusion is to be rate-controlled via a pump device. In any case, the infusion line must be flushed.^3^^,^^4^ This must be with the appropriate (compatible with the drug being administered) solution, at a minimal volume, above the residual volume of the line in use.^3^ On this point, to standardize practice whilst avoiding fluid overload, we recommend the flushing volume to be 20 mL.

To avoid non-compliance with line flushing of IV antimicrobial infusions, introduction of prompts on the prescription chart (paper or electronic) should be considered and, if possible, space to sign as confirmation that this has been done. We also recommend development and local implementation of total-dose policies for IV antimicrobial administration.

Broadening to wider recommendations, it is imperative that pharmaceutical and manufacturing companies ensure that instruction with regard to flushing of dose (if given by infusion) is explicit in their product recommendations. The manufacturers of infusion sets should also make reference to such instructions.^1^

Appraisal of options for various approaches to infusion-line flushing, including cost impact evaluation, would be required locally in each healthcare setting.^1^ A strong recommendation is made that national bodies should utilize and compare local determinations and use this to produce national guidelines on medicines management, optimization and safety, in this respect.

The most desirable and potentially ambitious recommendation is release of a National Patient Safety Alert to make all UK healthcare organizations aware of this under-recognized patient safety issue and make action compulsory. National Patient Safety Alerts are very helpful in ensuring organizational change is supported and would be much more effective than individual efforts to correct practice.^1^

As part of a National Patient Safety Alert, consensus on the terminology of IV line flushing should be communicated across all healthcare settings for standardization.^4^

There is much to do in terms of future research. This study has focused on IV administration of antimicrobials. However, these are not the only class of critical medicines. It is highly recommended that studies be undertaken with specific regard to critical medicines and ultimately conducted across all IV drug infusions.^4^^,^^9^

A national study of practice across all acute healthcare organizations to understand the level of awareness in the UK and which measures are being put in place to reduce drug dose losses would be beneficial.

It is also imperative to undertake a detailed investigation into the ethical and legal issues surrounding incomplete administration of a prescribed dose.^2^

Further research could also include looking into the limitations of this study and attempting to tackle specific groups of patients; for example, fluid-restricted patients, patients with renal impairment, paediatrics and patients at extremes of body weight.^6^

There may be some benefit in retrospectively researching the clinical impact of drug loss in terms of patient outcomes. This could include specific measures such as therapeutic dose monitoring, inflammatory and biochemical markers, as well as length of treatment, length of hospital stay and mortality rates.^2^ Such a study would be complex, with potential overlap or extension into researching the impact of lower doses on dose efficacy. This would need to look at MIC comparisons (actual measured or computer simulated) and time taken to reach therapeutic levels (especially for drugs requiring therapeutic drug monitoring).^9^

### Conclusions

This study demonstrates there can be significant drug loss when not flushing antibiotic infusion lines.^3^ The risk of deterioration in clinical condition, with very high potential for sepsis and mortality, warrant urgent action.^2^ Several recommendations are made to address this on a local and national level and to further the evidence base around this patient safety issue.^4^ The overall conclusion is that whilst further research will add to understanding the impact of such losses, administering the total dose of these critical medications should be made compulsory across all healthcare organizations, unless not doing so is clinically or legally justified, and this action should not be delayed.

## Funding

The study was carried out as part of our routine work.

## Transparency declarations

None to declare.
